# Shrinkage Mitigation of an Ultra-High Performance Concrete Submitted to Various Mixing and Curing Conditions

**DOI:** 10.3390/ma14143982

**Published:** 2021-07-16

**Authors:** Cédric Androuët, Jean-Philippe Charron

**Affiliations:** 1 Canadian Nuclear Safety Commission, 280 Slater Street, Ottawa, ON K1P 5S9, Canada; 2 Polytechnique Montréal, C.P. 6079, Succ. Centre-Ville, Montreal, QC H3C 3A7, Canada; jean-philippe.charron@polymtl.ca

**Keywords:** ultra high performance concrete, UHPC, UHPFRC, shrinkage, early-age behavior, curing

## Abstract

Ultra-High Performance Concretes (UHPC) are cement-based materials with a very low water-to-binder ratio that present a very-high compressive strength, high tensile strength and ductility as well as excellent durability, making them very interesting for various civil engineering applications. However, one drawback of UHPC is their pretty high autogenous shrinkage stemming from their very low water-to-binder ratio. There are several options to reduce UHPC shrinkage, such as the use of fibers (steel fibers, polypropylene fibers, wollastonite microfibers), shrinkage-reducing admixtures (SRA), expansive admixtures (EA), saturated lightweight aggregates (SLWA) and superabsorbent polymers (SAP). Other factors related to curing conditions, such as humidity and temperature, also affect the shrinkage of UHPC. The aim of this paper is to investigate the impact of various SRA, different mixing and curing conditions (low to moderate mixing temperatures, moderate to high relative humidity and water immersion) as well as different curing starting times and durations on the shrinkage of UHPC. The major importance of the initial mixing and curing conditions has been clearly demonstrated. It was shown that the shrinkage of the UHPC was reduced by more than 20% at early-age and long-term when the fresh UHPC temperature was closer to 20 °C. In addition, curing by water immersion led to drastic reductions in shrinkage of up to 65% and 30% at early-age and long-term, respectively, in comparison to a 20% reduction for fog curing at early-age. Finally, utilization of a liquid polyol-based SRA allowed for reductions of 69% and 63% of early-age and long-term shrinkages, respectively, while a powder polyol-based SRA provided a decrease of 47% and 35%, respectively.

## 1. Introduction

Ultra High Performance Concrete (UHPC), characterized by a very high compressive strength, high tensile strength and ductility, with a very low water-to-binder ratio, is becoming more and more prevalent in the construction field [1]. With excellent mechanical properties, UHPC can be used in several applications, such as high-rise buildings or bridges [2], precast structures [3,4] and strengthening of existing structures [5,6].

However, because of its very low water-to-binder ratio and very high quantity of binder, use of UHPC can be limited by its shrinkage behavior. High shrinkage is generally observed in UHPC specimens, resulting in a risk of cracking and deterioration [7]. Thus, serviceability, durability, as well as aesthetics of UHPC can be impaired [2,8]. In shrinkage development, the early-age shrinkage, which occurs shortly after casting of the specimen, is significant. Not only causing a concern to the dimension stability of UHPC elements, early-age shrinkage (especially when restrained) might generate micro cracks which, along with the aging of concrete, increase vulnerability of UHPC structures to substance penetration, leading to further deterioration [7,9].

Total shrinkage of concrete is mainly composed of autogenous shrinkage and drying shrinkage. The former is due to capillary depression caused by the hydration in the porous network and the latter is caused by evaporation of pore water to the surrounding. While the latter is the dominant factor in ordinary concrete shrinkage, it is the other way round for UHPC [10]. Since the water-to-binder ratio is very low in UHPC specimens, less pore water is available and transport mechanisms are very low for evaporation, thus drying shrinkage is minimized. In addition, in highly densified microstructure and fine pores of UHPC, according to Laplace’s Law, consumption of water by hydration lead to higher capillary pressure in the pore network, generating greater autogenous shrinkage [11]. Likewise, early-age autogenous shrinkage of UHPC can usually take up a significant part of the ultimate shrinkage value [12].

Various solutions can be used to reduce early-age shrinkage. The addition of fibers is usually adopted as an effective way. Steel fibers, polypropylene fibers (PPF) as well as wollastonite microfibers can mitigate shrinkage by either creating three-dimensional network or bridging effect between micro-cracks, while replacing part of the cement content to reduce hydration activity and self-desiccation. A hybrid effect is also observed while adding both steel fibers and PPF, leading to a better autogenous shrinkage control [13,14]. Microfibers are capable of transferring and redistributing stresses; they thus reduce crack width and favor multiple cracks in restrained shrinkage conditions [15].

Adding admixtures, such as shrinkage-reducing admixtures (SRA) or expansive admixture (EA), is also widely adopted. SRA is known to reduce surface tension at the gas-liquid interface of pores, leading to a decrease of capillary pressure, and thus mitigating the shrinkage. Some types of EA are capable of promoting ettringite formation, which results in an expansion behavior of the specimen. According to Yoo et al. [12], with addition of both admixtures, reduction in shrinkage was significant for both restrained and free shrinkage cases. Moreover, SRA has a retardant effect on the cement hydration process, while EA has proved to accelerate the hydration reaction [12,16].

Internal curing is another method to reduce shrinkage. This can be achieved by providing extra water from saturated lightweight aggregates (SLWA) or superabsorbent polymer (SAP). The former are saturated before being incorporated into the mix while the latter absorbs some of the mixing water. Both of them slowly release water continuously when capillary pressures develop. Adding SAP in UHPC can effectively reduce autogenous shrinkage and even induce an early-age expansion [17,18]. For mixtures incorporating SLWA, reduction of both the shrinkage rate and strain was observed, as well as a higher internal relative humidity [19,20].

Using industrial by-products may provide some shrinkage reduction as well. While autogenous shrinkage of concrete is generally increased when silica fume or ground granulated blast furnace slag are used [21,22,23,24], the use of fly ash tends to decrease the autogenous shrinkage of concrete [25,26].

Apart from the materials used in the UHPC mix-design, many other factors can also affect shrinkage and cracking behavior, especially curing conditions. Impact of curing conditions on UHPC was evaluated mainly for steam curing and the heat treatment process. In these specific conditions, acceleration of the hydration reaction and pore size reduction led to an increase of the shrinkage rate at early ages, while ultimate autogenous shrinkage value did not vary with different curing conditions [11,17,27]. However, information is lacking regarding effects of various field curing conditions on the shrinkage of UHPC, especially at early-age. More specifically, the effects of curing under moderate and high relative humidity, as well as under water immersion may result in a large variation of the UHPC shrinkage. Finally, the variation of curing starting time or curing duration can also have a significant impact on shrinkage development.

The purpose of this project is to evaluate the effects of different mixing and curing conditions on the shrinkage properties of a UHPC. Effects of various Shrinkage-Reducing Admixtures will be studied as well in order to be compared with the effects of mixing and curing conditions.

## 2. Materials and Methods

### 2.1. Materials

The experimental program was completed with a UHPC developed at Polytechnique Montreal [28], with 3 or 4% volumetric dosage of steel fibers. The material has a water-to-binder ratio of 0.2. The binder is made of cement and mineral admixtures. Other components are sand, 13-mm long straight smooth steel fibers of aspect ratio equal to 50 and a polycarboxylate-based superplasticizer. These UHPC have a characteristic compressive strength of 120 MPa at 28 days, which fulfills CSA A23.1:19 and CSA S6:19 requirements for Canadian UHPC.

Two different Shrinkage-Reducing Admixtures (SRA) composed of polyol-based additives were used to mitigate the shrinkage of UHPC. SRA1 is supplied in a liquid form, while SRA2 is provided in a powder form. This means SRA1 is added to the mix after water and superplasticizer are introduced, while SRA2 is mixed with dry components before introduction of liquid components.

### 2.2. Experimental Procedures

**Figure 1:**
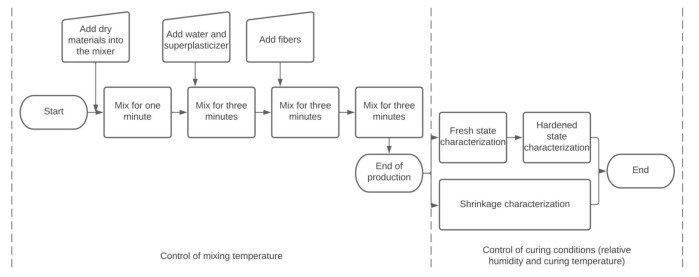
Sequences for the production and testing of the UHPC.

The sequences for the production and testing of the UHPC as well as periods of control of temperatures and relative humidity are presented in Figure 1. The mixing procedure consisted of mixing the dry components for one minute, adding water and superplasticizer during a mixing period of three minutes, adding fibers during a mixing period of three minutes and a last mixing stage of three minutes. This method provided a homogeneous and self-levelling UHPC with a total time of production shorter than other mixing procedures for UHPC [29,30].

The following tests were performed for all testing conditions: slump flow as per ASTM C1611 [31], mini-slump flow as per ASTM C1856 [32], air content of freshly mixed concrete as per ASTM C231 [33], temperature of freshly mixed concrete as per ASTM C1064 [34], compressive strength as per ASTM C39 [35] modified by ASTM C1856 [32], shrinkage as detailed below and continuous concrete temperature measurement for consideration of coefficient of thermal expansion in the analysis.

Shrinkage was measured by means of cylinders (height of 200 mm and diameter of 100 mm) with an embedded PMFL-50 strain gauge (50 mm length, uncertainty of ±7 μm/m) fixed in the center. The gauges were connected to a Campbell Scientific CR10 acquisition system powered by an Agilent E3616 DC power supply. All shrinkage results presented herein are average results obtained on two cylinders poured and cured in the same conditions. The difference in shrinkage measured between two specimens is on average less than 8%, varying between 0.7% and 11.9%. The smaller difference (0.7%) was obtained for the UHPC-F3% immersed in water at 9 h while the maximum difference of 11.9% was measured on the reference UHPC-F3%. No particular trend was observed regarding the variability of the test results vs. the various tested conditions, this variability being inherent to the material itself and the testing equipment used.

### 2.3. Experimental Program

The experimental program carried out to mitigate the UHPC shrinkage is detailed in Table 1. UHPC-F3% and UHPC-F4% mixes without SRA, mixed at 30 °C and cured at RH50% were considered reference materials (worst case scenario). The impact of the mixing temperature (10, 20 and 30 °C) was evaluated on the UHPC-F4% mix. Utilization of water at laboratory temperature (23 °C) allowed the production of the UHPC at 30 °C. Mixing temperatures at 20 °C and 10 °C were obtained by replacing water by 50% and 100% of ice, respectively. Mixing time with ice was increased by approximately 1 min per 50% of water replacement. The impact of a 7-day curing condition was evaluated with the UHPC-F3% mix. Casting of UHPC specimens into the molds was completed less than 30 min after contact between water and cement and then curing started at different periods after casting to reproduce different contexts happening in field conditions. Specific timeframes (9 h and 21 h) were selected to correspond to the times immediately before and after the Phase B of shrinkage curves, respectively (Section 3.2). Curing temperature was fixed at 20 °C, while relative humidity (RH) conditions were variable: 50%RH, 100%RH and fully submerged in water. The intent was to simulate a wide range of curing conditions, with RH50% as the worst case (with air curing), RH100% for fog curing and water immersion for continuous use of sprinklers (water in excess). After the first 7 days of curing, specimens were kept at RH50%. Finally, the effects of the addition of two different SRA were studied on the UHPC-F3%, both SRA1 and SRA2 being tested with a dosage of 2% by weight of cementitious materials.

## 3. Results

### 3.1. Fresh State and Hardened State Properties

The fresh state and hardened state properties of the UHPC produced in this project are summarized in Table 2. All UHPC mixtures showed a mini-cone slump flow between 216 and 232 mm and a compressive strength at 28 days between 123 and 131 MPa. Using SRA or varying the mixing temperatures did not have any significant impact on the fresh state properties nor on the UHPC compressive strength measured at 28 days.

### 3.2. Data Treatment and Analysis of Early-Age Shrinkage

**Figure 2:**
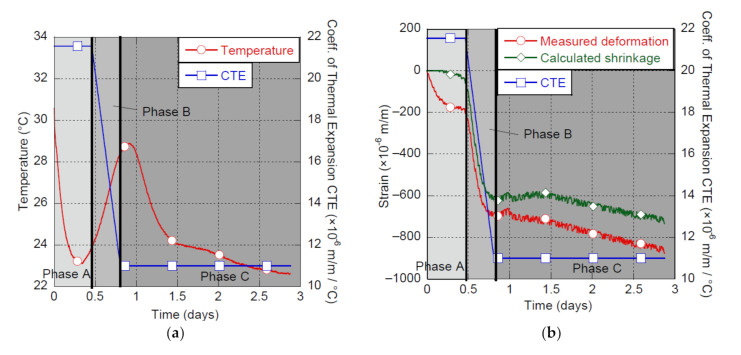
Early-age behavior of the UHPC-F3%, (**a**) temperature and CTE vs. time, (**b**) shrinkage and CTE vs. time.

Early-age deformation of UHPC (first 24 h after contact between water and cement) has a very particular behavior that can be separated in three different phases as illustrated in Figure 2 on the reference UHPC-F3% mix without SRA, produced at 30 °C and cured at a relative humidity of 50%. The analysis procedure described further was applied to all mixes studied.

In Phase A, a negative deformation (contraction) of approximately 190 μm/m occurs during the first 10 to 12 h. Then Phase B shows a prompt increase in negative deformation with a magnitude around 500 μm/m, from 12 to 20 h approximately. In Phase C, a slight swelling occurs, followed by a progressive increase of the negative deformation that will continue at long term.

Distinction must be made between what is called negative deformation and shrinkage of the material itself. Indeed, deformation measured at very early age considers not only shrinkage of the material but thermal deformation as well, because of the hydration heat of the binder. It is noteworthy to mention that even in a temperature-controlled environment for curing, specimens undergo a small temperature variation, particularly for UHPC containing high binder content. In that context, the shrinkage can be computed once the thermal deformation is removed from the specimens’ deformation. To do so, the coefficient of thermal expansion (CTE), which varies at early age [36,37,38], must be defined.

Negative deformation developed during Phase A is mainly due to temperature differences between concrete and ambient air [10,39]. The CTE initial value of 21.6 μm/m/°C was computed considering negative deformation developed during the first five hours of life of the material. During Phase B, a linear reduction of the CTE is a good approximation of its real behavior [37]. The CTE final value is reached at the beginning of Phase C and proposed equal to 11 μm/m/°C for UHPC [40,41]. CTE evolution for the UHPC tested is presented in Figure 2a, while the analysis of early-age deformation of the UHPC is shown in Figure 2b. In this example the data treatment removed approximately 150 μm/m of thermal deformation, which is significant. Moving forward, shrinkage results will be presented after being processed for temperature effects as detailed above. As mentioned in the Introduction, the UHPC shrinkage is mainly related to autogenous shrinkage, itself caused by consumption of pore water in the concrete inner structure by hydration [10].

During Phase B, a very sharp increase of shrinkage is observed. UHPC particles get increasingly closer as the material is switching between a plastic state and a state where plastic rearrangements are impeded by the increasing stiffness of the matrix [42,43]. This rigidity is proportional to mechanical links formed between particles [44]. This phase is most surely related to the mineral percolation threshold identified by [42].

Shrinkage in Phase C occurs at a much lower pace than in Phase B and is counterbalanced initially by a slight swelling. The swelling is generally associated with ettringite and calcium hydroxide crystals formation [42,45,46,47] as well as topochemical reaction of C_3_S [42,48]. The decrease in shrinkage kinetics in Phase C is due to the result of restraint caused by hydrates development in the concrete matrix [12,42] and the resistance to the contraction due to capillary depression [49].

It is noteworthy that the magnitude of shrinkage shown in this paper is high due to several reasons. Especially, the shrinkage measurements from the casting time are presented, while most publications show shrinkage from demolding or after 24 h of production. As can be seen in Figure 2, up to 700 μm/m would be neglected if the shrinkage measurements were started 24 h after contact between water and cement. Because mixing and curing conditions as well as SRA addition have a strong impact on this very early-age shrinkage, it was important to analyze the shrinkage behavior starting from the casting time.

### 3.3. Effect of Mixing Temperature on Shrinkage

**Figure 3:**
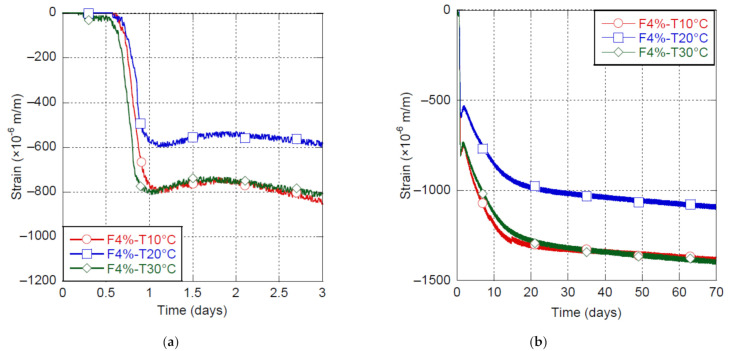
Shrinkage of UHPC-F4% at 10, 20 and 30°C mixing temperatures, (**a**) early-age shrinkage, (**b**) long-term shrinkage.

The shrinkage of the UHPC-F4% is presented in Figure 3 for mixing temperatures of 10, 20 and 30 °C.

Decreasing mixing temperature from 30 °C to 20 °C allowed lowering Phase B shrinkage from around 800 μm/m to 600 μm/m, which is a reduction of 25%. Decreasing mixing temperature up to 10 °C delayed setting time of the concrete, but did not provide a supplementary reduction of shrinkage. The shrinkage measured at 10 °C and 30 °C were of the same magnitude. A similar trend was also observed by [50] for ordinary concrete. They proposed that low initial temperature may increase the viscosity of pore water and the stress at the gas-liquid interface and thus favor shrinkage. The impact of the mixing temperature at early age remained in the long term. Shrinkage measured at mixing temperatures of 10 °C and 30 °C were equivalent at 70 days of age, while it was reduced by about 25% at a mixing temperature of 20 °C. This impact of mixing temperatures was also observed in UHPC mixtures produced with SRA (results are not presented in this paper).

### 3.4. Effect of Humidity Conditions on Shrinkage

#### 3.4.1. RH100% in a Fog Room

**Figure 4:**
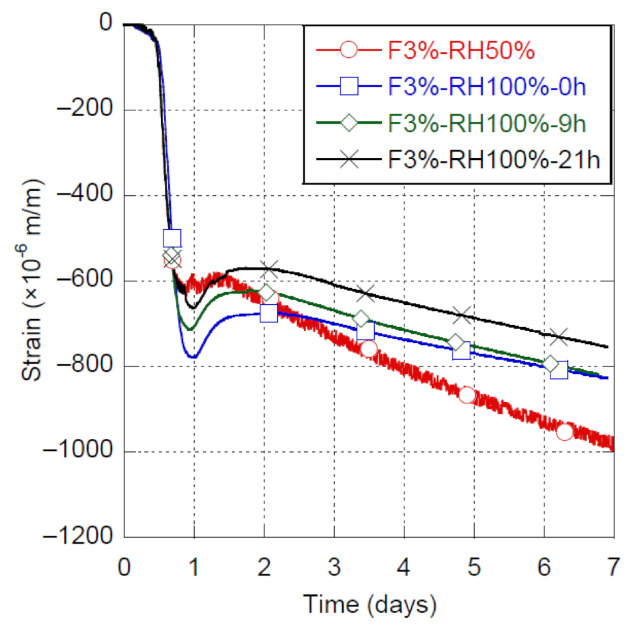
Shrinkage of UHPC-F3% with RH100% started at 0, 9 and 21 h.

The shrinkage of the UHPC-F3% is presented in Figure 4 for curing conditions at 20 °C and RH100% started at 0, 9 and 21 h, the reference condition at RH50% is also shown. The behavior of the mix submitted to the different curing conditions is the same during Phase A controlled by thermal variation. Only a slight difference exists in early-age shrinkage during Phase B; this difference is of similar magnitude to the one observed between twin specimens poured and cured in the same condition. Finally, evolution of deformation observed in Phase C is very similar for all fog curing conditions presented in Figure 4. It is interesting to note that counterintuitively, no matter when the RH100% curing condition starts in the first 24 h following casting, equivalent early-age shrinkage were observed in the first 7 days.

In comparison to the reference condition (RH50%), fog curing (RH100%) shows shrinkage slightly higher in Phase B, but significantly lower in Phase C. Fog curing favors a greater swelling at the beginning of Phase C, probably due to higher availability of water for ettringite formation. It is worth noting that the swelling observed at the beginning of Phase C fully counterbalances the slight increase in shrinkage observed during Phase B. Finally, fog curing provides afterwards a shrinkage reduction of about 20% at 7 days, by filling pores partially emptied by hydration.

#### 3.4.2. Immersion in Water

**Figure 5:**
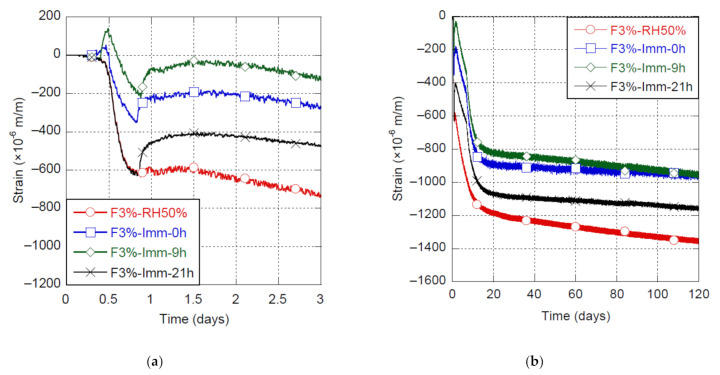
Shrinkage of UHPC-F3% with immersion started at 0, 9 and 21 h, (**a**) early-age shrinkage, (**b**) long-term shrinkage.

The shrinkage of the UHPC-F3% is presented in Figure 5 for curing conditions at 20 °C and immersion in water starting at 0, 9 and 21 h, the reference condition at RH50% is also shown. Moreover, the shrinkage occurring in Phase B, the swelling occurring at the beginning of Phase C and the combined shrinkage and swelling at 1, 7 and 120 days are detailed in Table 3.

Immersion of specimens into water generated an immediate swelling, no matter when the specimens were immersed (0, 9 and 21 h). This is due to physical water absorption between particles and hydrates as well as filling of the partially empty pores. Phase B shrinkage was approximately 580 μm/m with immersion at 21 h or without immersion (RH50%). When immersion occurred at 0 and 9 h (i.e., before the beginning of Phase B), Phase B shrinkage was greatly reduced to 390 and 335 μm/m, respectively. Immersion before the beginning of Phase B allowed for a reduction of shrinkage occurring during Phase B of up to 40%. This is most likely due to the fact that, at such an early age, porosity within the material is still opened and connected, which allows water to fill it in. This allows for a more gradual variation of pore pressure caused by hydration and drastically decreases the shrinkage intensity.

Moreover, the swelling that occurred at the beginning of Phase C was notably amplified when the specimens were immersed in water (Figure 5). Indeed, Phase C swelling was about 175 ± 15 μm/m for specimen under immersion, while it was only 20 μm/m for UHPC continuously kept at RH50%. This can be due to the greater availability of water for the hydration process of C_3_A and C_4_AF, which requires a large amount of water [51]. Hydration of C_3_A and C_4_AF leads to the formation of ettringite [51], which is mainly responsible for the swelling observed at the beginning of Phase C [42,45,46,47]. In comparison to the RH50% condition, this led to a reduction of shrinkage at 7 days of 50%, 65% and 34% for specimens immersed at 0, 9 and 21 h, respectively.

Figure 5b shows the change in shrinkage slope of immersed specimens happening at 7 days, when they were removed from water and submitted to RH50%. Indeed, shrinkage increase between 7 and 20 days was approximately 410 ± 10 μm/m, while it was about 200 μm/m for UHPC continuously kept at RH50%. Even if the shrinkage increased significantly when specimens were removed from water, mid-term shrinkage measured since UHPC production was smaller than for specimens that did not experience immersion. Moreover, as strength and rigidity of the material are much higher at 7 days than at early-age, material is then able to withstand those strain, unlike when happening at early-age.

Furthermore, long-term shrinkage kinetics was lower when specimens have been immersed in water, no matter when the immersion occurred. According to Figure 5b, water curing by immersion allowed for a reduction of shrinkage of up to 75% at 4 days and around 30% at 120 days. This most significant shrinkage reduction happened when specimens were submerged before Phase B starts. More specifically, submerging specimens just before Phase B starts (at 9 h of age) allowed keeping shrinkage/swelling strain between +130 μm/m and −220 μm/m until reaching more than 4 days of life of the material. In comparison with a shrinkage strain of 620 μm/m at 20 h and of 800 μm/m at 4 days for the mix under the reference conditions, this clearly strengthens the major importance of water curing at very early-age for controlling the shrinkage behavior of UHPC.

### 3.5. Effect of SRA on Shrinkage

**Figure 6:**
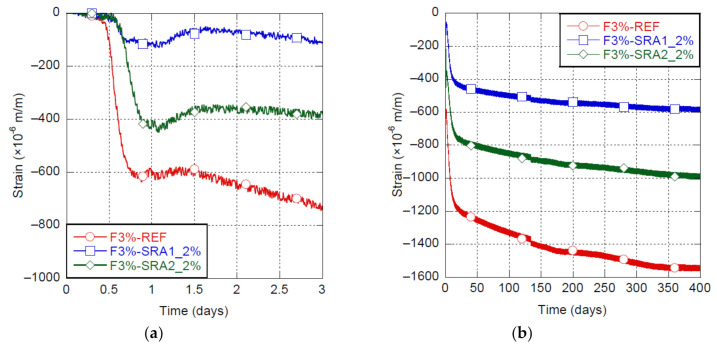
Shrinkage of UHPC-F3% with and without SRA, (**a**) early-age shrinkage, (**b**) long-term shrinkage.

Shrinkage of the UHPC-F3% mix with and without SRA is illustrated in Figure 6. Furthermore, the shrinkage occurring in Phase B, the swelling occurring at the beginning of Phase C and the combined shrinkage and swelling at 1, 7 and 365 days are detailed in Table 4.

These mixes were produced at 30°C and kept continuously at RH50%. The SRA1 allowed for a better control of early-age shrinkage happening during Phase B, with a very significant reduction of shrinkage at 1 and 7 days of 81% and 69%, respectively, comparatively to 27% and 43% for the SRA2, respectively. This reduction of shrinkage is related to the reduction of the surface tension at the gas-liquid interface in capillary pores provided by SRA. Additionally, the swelling at the beginning of Phase C was increased from 20 μm/m for the reference mix to 65 μm/m for mixes with SRA. During Phase B and Phase C, deformation is a combination of shrinkage and swelling (related to ettringite, calcium hydroxide crystals formation and topochemical reaction of C_3_S). It is likely that the reduction of shrinkage allowed for a better observation of the swelling, probably not impacted by the introduction of SRA.

At long-term, the SRA1 provided a shrinkage reduction of 63% rather than 35% for the SRA2. For this project, the SRA1 in liquid form was clearly more efficient to mitigate the UHPC shrinkage.

## 4. Discussion

Early-age behavior of UHPC is of major importance as more than half of the shrinkage observed at 28 days occurs during its first 24 h of maturity. In that context, a procedure was developed to accurately evaluate the UHPC shrinkage from the casting time by removing the thermal deformation. Different parameters were investigated in this project to get a better understanding and control of the UHPC shrinkage.

Environmental conditions during mixing and curing of UHPC can have a major impact on early-age and long-term shrinkages of UHPC. A mixing temperature close to 20 °C is optimal to reduce about 25% of early-age (7 days) and long-term shrinkages, as compared to 10 and 30 °C. This impact of temperature cannot be explained solely by the effect of temperature on UHPC maturity, it is probably also related to modification in water viscosity in pores at lower temperature and to surface tension magnitude at gas-liquid interface in the pores [50]. Whatever the temperature at the construction site, it seems preferable for mitigating shrinkage to adjust the UHPC mixing temperature around 20 °C, by controlling UHPC components temperature for example.

Humidity conditions during curing modify significantly the magnitude of shrinkage. In this project, curing conditions were applied immediately after UHPC casting in molds was finished. In comparison to RH50%, curing carried out in a fog room (RH100%) decreases by 20% the early-age shrinkage (7 days). Curing at RH100% appears to be much less efficient in reducing early-age shrinkage than curing by immersion under water.

Curing with water immersion reduces very significantly the UHPC shrinkage, up to 65% at early ages (7 days) and 30% at long term. While fog curing avoids water evaporation from the UHPC matrix to the surroundings and should provide little water to fill pores partially emptied by hydration, water immersion provides much more water to efficiently fill pores and favor water adsorption between hydrates. Therefore, water immersion causes an additional swelling during Phase B by water adsorption and magnifies swelling at the beginning of Phase C, mainly by ettringite formation. Based on this experimental program, it appears optimal to start the curing by immersion at 9 h, just before Phase B starts, to greatly reduce early-age and long-term shrinkages. It is not recommended to apply the water curing before to avoid any eventual washout of the surface in contact with the curing water. Water immersion can be achieved with sprinklers providing water on the UHPC surface.

Using polyol-based SRA in liquid form at a 2% dosage allowed a reduction of early-age (7 days) and long-term shrinkages of UHPC of up to 69% and 63%, respectively, with a substantial reduction of 85% at 3 days. The same SRA in powder form provided lower reduction of shrinkage of 43% and 35% at early age (7 days) and long term, respectively. These reductions of shrinkage are related to the reduction of the surface tension at the gas-liquid interface in capillary pores provided by the SRA.

Cumulating both SRA and immersion of UHPC could be of great interest in reducing even more UHPC early age shrinkage and thus reducing related issues detailed in the Introduction. Moreover, allowing a longer curing period may further reduce the shrinkage strain.

## 5. Conclusions

Because of its very low water-to-binder ratio and high binder content, Ultra-High Performance Concrete (UHPC) generally present high shrinkage, especially at early ages. High early-age shrinkage can lead to early-age cracking and hence potentially affect serviceability, durability, as well as aesthetics [2,7,8]. Various options for mitigating early-age shrinkage of UHPC have been considered in this research project, including the use of SRA as well as various mixing temperatures and curing conditions. Based on the experimental results, the following conclusions can be drawn:

- A mixing temperature of 20 °C allowed a decrease of 25% of early-age (7 days) and 21% of long-term UHPC shrinkage, in comparison to 10 and 30 °C;
- Curing UHPC by water immersion greatly reduced UHPC shrinkage up to 65% at early age (7 days) and 30% at long term, while curing at RH100% provided a 20% reduction at 7 days. The best control of early-age and long-term shrinkages was obtained by curing with immersion when it started at 9 h, just before early-age deformation entered its Phase B. This allowed to maintain the deformation due to swelling and shrinkage between +130 μm/m and −220 μm/m until reaching more than 4 days of maturity in comparison with a shrinkage strain of 620 μm/m at 20 h and of 800 μm/m at 4 days for the mix under the reference conditions;
- The use of polyol-based SRA in liquid form at a 2% dosage allowed to reduce by 69% and 63% early-age (7 days) and long-term shrinkages of UHPC, respectively, whereas a reduction of 43% and 35%, respectively, was observed when SRA was introduced in powder form at the same dosage.

The combination of shrinkage mitigation solutions considered in this project (SRA, 20 °C mixing temperature and curing by immersion) could be of great interest in order to minimize restrained shrinkage cracking of UHPC in structures.

## Figures and Tables

**Table 1:** Experimental program.

Parameter	Material	SRA Type	Mixing Temperature	Curing Type and Starting Time ^1^	
Temperature	UHPC-F4%	None	10 °C	RH50%	0 h after casting
			20 °C		
			30 °C		
Humidity	UHPC-F3%	None	30 °C	RH50%	0 h after casting
				RH100%	0, 9 or 21 h after casting
				Immersed	0, 9 or 21 h after casting
SRA	UHPC-F3%	SRA1—2%	30 °C	RH50%	0 h after casting
		SRA2—2%			

^1^ Characteristics of the first 7 days of curing, afterwards curing at RH50%.

**Table 2:** Fresh state and hardened state properties.

Material	Condition	Slump Flow (Mini-Cone/Abrams Cone)	Air Content	Temperature	Compressive Strength (28 d)
UHPC-F3%	No SRA	231 / 720	2.9	31.4	125.4
	SRA1—2%	228 / 710	2.7	30.9	124.6
	SRA2—2%	232 / 720	3.1	31.1	123.1
UHPC-F4%	Mixing 10 °C	229 / 710	3.2	10.4	130.6
	Mixing 20 °C	228 / 710	2.9	20.6	131.0
	Mixing 30 °C	216 / 700	3.1	30.4	128.9

**Table 3:** Effect of the immersion on the shrinkage and swelling of the UHPC-F3%.

Material	Condition	Shrinkage in Phase B	Swelling (at the Beginning of Phase C)	Combined Shrinkage and Swelling		
				at 1 d	at 7 d	at 120 d
UHPC-F3%	RH50%	580 μm/m	20 μm/m	596 μm/m	995 μm/m	1367 μm/m
	Immersion at 0 h	390 μm/m	160 μm/m	224 μm/m	495 μm/m	965 μm/m
	Immersion at 9 h	335 μm/m	170 μm/m	80 μm/m	345 μm/m	949 μm/m
	Immersion at 21 h	580 μm/m	190 μm/m	459 μm/m	656 μm/m	1157 μm/m

**Table 4:** Effect of SRA on the shrinkage and swelling of UHPC-F3%.

Material	Condition	Shrinkage in Phase B	Swelling (at the Beginning of Phase C)	Combined Shrinkage and Swelling		
				at 1 d	at 7 d	at 365 d
UHPC-F3%	No SRA	580 μm/m	20 μm/m	596 μm/m	995 μm/m	1540 μm/m
	SRA1_2%	100 μm/m	65 μm/m	115 μm/m	307 μm/m	571 μm/m
	SRA2_2%	395 μm/m	65 μm/m	435 μm/m	566 μm/m	996 μm/m

## Data Availability

Not applicable.
